# Enhancing Physiochemical Substrate Properties of Thin-Film Composite Membranes for Water and Wastewater Treatment via Engineered Osmosis Process

**DOI:** 10.3390/polym15071665

**Published:** 2023-03-27

**Authors:** Wan Nur Ain Shuhada Abdullah, Nadiene Salleha Mohd Nawi, Woei Jye Lau, Yeek Chia Ho, Farhana Aziz, Ahmad Fauzi Ismail

**Affiliations:** 1 Advanced Membrane Technology Research Centre (AMTEC), Universiti Teknologi Malaysia, Skudai 81310, Malaysia; 2 Civil and Environmental Engineering Department, Universiti Teknologi PETRONAS, Seri Iskandar 32610, Malaysia; 3 Centre for Urban Resource Sustainability, Institute of Self-Sustainable Building, Universiti Teknologi PETRONAS, Seri Iskandar 32610, Malaysia

**Keywords:** substrates, coating, TFC membrane, AT-POME, graphene oxide

## Abstract

The commercial thin-film composite (TFC) nanofiltration (NF) membrane is unsuitable for engineered osmosis processes because of its thick non-woven fabric and semi-hydrophilic substrate that could lead to severe internal concentration polarization (ICP). Hence, we fabricated a new type of NF-like TFC membrane using a hydrophilic coated polyacrylonitrile/polyphenylsulfone (PAN/PPSU) substrate in the absence of non-woven fabric, aiming to improve membrane performance for water and wastewater treatment via the engineered osmosis process. Our results showed that the substrate made of a PAN/PPSU weight ratio of 1:5 could produce the TFC membrane with the highest water flux and divalent salt rejection compared to the membranes made of different PAN/PPSU substrates owing to the relatively good compatibility between PAN and PPSU at this ratio. The water flux of the TFC membrane was further improved without compromising salt rejection upon the introduction of a hydrophilic polydopamine (PDA) coating layer containing 0.5 g/L of graphene oxide (PDA/GO0.5) onto the bottom surface of the substrate. When tested using aerobically treated palm oil mill effluent (AT-POME) as a feed solution and 4 M MgCl_2_ as a draw solution, the best performing TFC membrane with the hydrophilic coating layer achieved a 67% and 41% higher forward osmosis (FO) and pressure retarded osmosis (PRO) water flux, respectively, compared to the TFC membrane without the coating layer. More importantly, the coated TFC membrane attained a very high color rejection (>97%) during AT-POME treatment, while its water flux and reverse solute flux were even better compared to the commercial NF90 and NF270 membranes. The promising outcomes were attributed to the excellent properties of the PAN/PPSU substrate that was coated with a hydrophilic PDA/GO coating and the elimination of the thick non-woven fabric during TFC membrane fabrication.

## 1. Introduction

Water shortages and deterioration of water quality are the main concerns worldwide in the 21st century. Significant attention has been paid to wastewater treatment and reuse as a sustainable solution to address these issues. Malaysia is now the second largest crude palm oil (CPO) producer after Indonesia, contributing 39% of world palm oil production and 44% of world exports [1]. Despite the fact that the palm oil industry contributed significantly to the Malaysian economy and improved the living standard of local society [2], the discharge of large amounts of oily wastewater from this industry remains the main concern to the public.

The effluent discharged by the palm oil refining process attracts a great deal of attention mainly due to its large volume produced yearly, i.e., >3.86 million tons of palm oil mill effluent (POME) [3,4]. This effluent generally contains a high concentration of organic pollutants. Such wastewater, if discharged into receiving water bodies without proper treatment processes, could contaminate water and affect the ecosystem. Currently, the biological-based treatment process is the most commonly employed method in the industry to treat the POME by significantly reducing the level of chemical oxygen demand (COD), biological oxygen demand (BOD), and total suspended solid (TSS) [5]. However, it is not effective to completely remove organic pollutants from wastewater [6].

Over the past decade, the employment of the membrane technology for industrial water and wastewater treatment has been growing steadily. A market report indicated that the market size of the membrane is projected to reach USD 8.3 billion by 2024 from USD 5.4 billion in 2019 [7]. The major drivers for the significant market growth are rising awareness about wastewater recycling and rapid industrialization coupled with a growing population. A promising separation efficiency could be achieved by employing suitable membrane properties [8,9]. The microporous ultrafiltration (UF) membrane was found to be able to produce a high water flux while treating aerobically treated palm oil mill effluent (AT-POME), but the nano-sized pigments were not effectively rejected, as they still presented in the permeate [10]. For the denser nanofiltration (NF) membrane, a study showed that it encountered a higher degree of fouling during the treatment process, even though the membrane could produce high-quality permeate [10]. In view of this, an emerging osmotically driven membrane process (i.e., forward osmosis (FO)/pressure retarded osmosis (PRO)) that requires no external driving force but can still remove pollutants from a wastewater source becomes the promising candidate. It must be pointed out that the typical reverse-osmosis (RO)-like FO membranes for brackish water or seawater desalination applications might not be suitable for wastewater treatment, owing to its dense selective layer that exhibits high water transport resistance and low water permeability.

Thus, NF-like FO membranes can be potentially used together with draw solutions made of either divalent salts or polyelectrolytes where water from feed solution permeates through the membrane while contaminants in the feed solution are retained by the membranes. The rationale on the use of a wastewater sample that has been aerobically treated on-site in the industry (known as AT-POME) is because of the difficulty of the current technologies in removing phenolics and tannins from the AT-POME. The presence of both compounds makes the effluent not only brownish but also unfit for reuse [11]. In 2018, our group demonstrated that a commercial thin-film composite (TFC) NF membrane (supported by nonwoven fabric) could be used to treat AT-POME under the FO/PRO process [12], but the top surface characteristics of the TFC membrane and its substrate need to be further optimized to achieve higher water permeability.

Thus, in the present work, we develop a TFC membrane consisting of a polyamide (PA)-selective dense layer and hydrophilic polyacrylonitrile/polyphenylsulfone (PAN/PPSU) porous support layer in the absence of thick nonwoven fabric, aiming to achieve higher membrane permeance and better antifouling properties during the osmotically driven process. PAN substrates are well known for their low fouling character for aqueous filtrations due to their hydrophilic nature [13]. PPSU, on the other hand, has superior properties compared to the frequently used polysulfone. It has a higher heat resistance, long-term thermal stability, and broad range of chemical compatibility, which make it a remarkable candidate for the synthesis of an ideal substrate for TFC membranes [14]. Polymers blending is always adopted by researchers for the microporous membranes fabrication as it can take the advantages of both physical and chemical features of each polymer to achieve a synergetic effect [15,16,17].

Apart from the optimization of the PA-selective layer, utilization of a hydrophilic support layer is critical to improve the water flux of the TFC NF membrane by enhancing water diffusion across the membrane [18,19]. The TFC NF membrane with chemical modification at the bottom layer is believed to be able to further improve the properties and performances of the TFC NF membrane for the engineered osmosis process. In this work, the TFC NF membrane is further improved by coating its substrate bottom with polydopamine/graphene oxide (PDA/GO), aiming to improve its hydrophilicity and antifouling tendency during AT-POME treatment. The PDA coating has been widely used to increase membrane permeability due to hydrophilicity while GO shows a strong potential to achieve an excellent membrane performance in terms of water flux, rejection of target chemicals, and fouling resistance [20,21,22,23,24].

The main objective of this work is to develop a NF-like FO/PRO membrane for effective treatment of AT-POME. In the first part of the work, different substrates are developed for the NF-like FO/PRO membrane fabrication by varying the weight ratio of PAN and PPSU in the dope solution. In the second part of the work, the bottom surface of PAN/PPSU is further modified through an additional coating layer composed of PDA and GO. Such a hydrophilic coating layer aims to reduce the membrane water transport resistance without affecting selectivity. It is also believed that the hydrophilic PDA/GO layer could play a key role in enhancing the TFC NF membrane with respect to reverse solute flux and offer a good solution to treat AT-POME in the engineered osmosis process.

## 2. Methodology

### 2.1. Materials

To fabricate the microporous substrate for the TFC membrane, polyacrylonitrile (PAN) (molecular weight (MW): 10,000 g/mol) and polyphenylsulfone (PPSU) (MW: 50,000 g/mol) purchased from Sigma-Aldrich (St. Louis, MO, USA) and Solvay Advanced Polymers (Greenville, SC, USA) were used, respectively. To prepare the dope solution, 1-methyl-2-pyrrolidone (NMP) with 99% purity from Acros Organics (Geel, Belgium) was used as solvent to dissolve PAN and PPSU. In order to establish the PA layer atop the PAN/PPSU substrate, piperazine (PIP) and trimesoyl chloride (TMC) obtained from Acros Organics (Geel, Belgium) were, respectively, used as the active amine monomer and acyl chloride monomer. GO was synthesized according to the method as described in our previous work [25]. Dopamine hydrochloride with 98% purity and tris-hydrochloride (Tris-HCl) with 99% purity purchased from Sigma-Aldrich (St. Louis, MO, USA) were utilized to prepare PDA/GO solution and were used as coating materials for the bottom surface of the PAN/PPSU substrate. Magnesium chloride (MgCl_2_), magnesium sulfate (MgSO_4_), and sodium sulfate (NaSO_4_) obtained from Sigma-Aldrich (St. Louis, Missouri, USA) were used as either feed solutes in the NF system or draw solutes in the osmotically driven process by dissolving them separately in pure water to prepare solutions with the desired solute concentration. Two commercial NF membranes, i.e., NF90 and NF270, supplied by DuPont FilmTec^TM^ (Edina, MN, USA) in dry condition were also tested during the FO/PRO process, and the results were used to compare with the performance of the membranes developed in this study.

The AT-POME samples were obtained from the PPNJ Palm Oil Mill Kahang located in Johor and stored at 4 °C prior to use. As there is no specific standard for the color determination of AT-POME, the commonly used methods in determining the color of water samples were, therefore, adopted in this study. The effluent samples were characterized with respect to conductivity, color, and total organic carbon (TOC). The conductivity of the sample was measured by a benchtop conductivity meter (4520, Jenway, London, UK), while a UV-vis spectrophotometer (DR5000, Hach, Singapore) and TOC analyzer (TOC LCPN, Shimadzu, Japan) were used to determine the color and TOC, respectively. Table 1 summarizes the characteristics of the AT-POME used in this work.

### 2.2. Fabrication of Microporous Substrate

To prepare the dope solution for the substrate, pre-weighted PAN pellets were first dissolved in NMP. Once they were fully dissolved, PPSU pellets were gradually introduced into the mixture. The quantity of respective polymer added was based on the PAN/PPSU ratio, as stated in Table 2. In total, both polymers accounted for 16 wt% of the total weight of the dope solution. The prepared dope solution was continuously stirred at 60 °C for 24 h to produce homogeneous solution. The solution was then left overnight without stirring to eliminate any gas bubbles trapped in the solution. The dope was then poured on a dry glass plate followed by casting using a glass rod with its film thickness controlled at 110 ± 5 μm. The cast substrate was left for 10 s at ambient temperature before immersing into a water coagulation bath at room temperature to initiate the phase inversion process. Once the membrane was peeled off from the glass plate, it was transferred into another water bath and kept for 24 h to remove residual solvent. Afterward, the substrate was soaked in 2 M NaOH solution for 40 min for hydrolysis. Finally, the resultant substrate was washed with reverse osmosis water and maintained in wet conditions prior to use.

### 2.3. Fabrication of Polyamide Layer

The PA-selective layer of TFC membranes was prepared via in situ interfacial polymerization of PIP and TMC atop the fabricated substrates. The substrate (with skin layer facing up) was first clamped in between a glass plate and Viton frame. An amount of 30 mL of 2 *w*/*v*% PIP aqueous solution was then poured on top of the PAN/PPSU substrate and was held for 2 min before draining off the excess solution. The residual water droplets were rolled off from the amine-saturated substrate using a commercial soft rubber roller. Then, 30 mL of 0.2 *w*/*v*% TMC in n-hexane was poured onto the PIP-saturated substrate surface to initiate cross-linking. The solution was drained off from the surface after 1 min of contact. The interaction between two active monomers resulted in the formation of a thin active PA layer over the substrate, forming the TFC membrane. Afterward, the TFC membrane was oven-dried for 3 min at 60 °C. Finally, the TFC membrane was washed thoroughly with RO water and stored in RO water at 5 °C prior to use. The procedure of developing the PA layer is the same for all types of TFC membranes except a different PAN/PPSU substrate was used during the interfacial polymerization process.

### 2.4. Fabrication of Coating Layer

**Figure 1:**
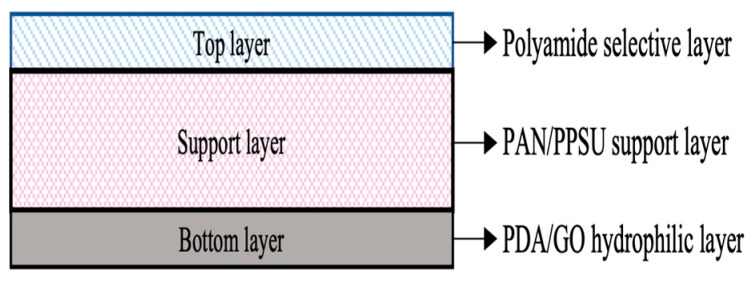
Schematic illustration of TFC membrane with PDA/GO coating layer. Note: Not to scale.

An additional layer was formed on the bottom surface of the substrate of the TFC membrane via self-polymerization of PDA. First, 0.1 g of dopamine hydrochloride was dissolved in 50 mL of DI water. Then, 1 mL of tris-HCl was added in the solution to adjust the solution pH to ~8.5. Different concentrations of GO aqueous solution (in 5 mL) were then added into the 50 mL PDA solution (20 g/L) followed by 3 h of ultra-sonication to properly disperse GO. The concentration of the GO in water was varied from zero to 0.7 g/L. Next, the bottom surface (facing up) of the TFC membrane was first clamped in between a glass plate and Viton frame. An amount of 30 mL of PDA/GO solution was poured on the bottom surface and was held for 1 h on a shaker before draining off the excess solution. After removal of excess solution, the surface was water-rinsed to terminate the reaction and stored in DI water. Figure 1 shows the schematic illustration of the TFC membrane composed of three important layers, i.e., thin PA-selective layer, PAN/PPSU substrate, and PDA/GO coating layer. The membrane was labeled as PDA/GO-0.3, PDA/GO-0.5, and PDA/GO-0.7, depending on the concentration of GO used in coating solution.

### 2.5. Characterization of Membranes

A field emission scanning electron microscope (FESEM) (Crossbeam 340, Zeiss, Birmingham, UK) was used to study the surface morphology and cross-sectional structure of the PAN/PPSU substrate as well as the TFC membrane at different magnifications. Fourier transmission Infrared spectroscope-attenuated total reflectance (FTIR-ATR) (Avatar 360, Thermo Nicolet, Thermo Fisher Scientific, Waltham, MA, USA) was utilized to identify the functional groups of the substrate and PA-selective layer. Each FTIR spectrum was collected between 4000 and 500 cm^−1^ and the results were the average of 32 scans. The Omnic software was used to process acquired data. An atomic force microscope (AFM) (NX10, Park Systems, Suwon, Republic of Korea) was utilized to obtain the mean roughness of the membrane at a scan area of 10 μm × 10 μm. The surface hydrophilicity meanwhile was measured using a contact angle (CA) goniometer (OCA 15Pro, DataPhysics, Filderstadt, Germany). The volume of pure water dosing was fixed at 0.3 μL when it was dropped on a dried membrane surface.

### 2.6. Performance Evaluation of Membranes

Prior to the FO/PRO filtration experiment, the water flux and solute rejection of membranes were first evaluated using a pressure-driven filtration setup composed of a 320 mL dead-end permeation cell (HP4750, Sterlitech, Auburn, WA, USA). All the membranes were subjected to a 30 min compaction at 11 bar to achieve a stable condition. Afterward, the pure water flux of the membrane was determined at 10 bar. In order to identify the suitable solutes that can be used as draw solutes during the FO/PRO process, the water flux and rejections of membranes in filtering different solutes were determined. The rejections of divalent salt (Na_2_SO_4_ or MgSO_4_) need to be at least 90% as it is one of the criteria to confirm the membrane properties (NF category). A solution containing 1000 ppm (equivalent to mg/L) of single solute was prepared and used as feed for the filtration process. The water flux and rejection were determined and collected for a period of 30 min for each membrane. Upon completion of the experiment, the membrane was further tested with AT-POME. Average water flux and rejection were reported for each membrane for each set of experiments.

The membrane pure water flux, *J*, and water permeability, *A*, were measured using Equations (1) and (2), respectively.

(1) $$J=\frac{\Delta V}{A_{m}\cdot \Delta t}$$

(2) $$A=\frac{J}{\Delta P}$$

where ∆*V* is the volume of permeate water flux, *A_m_* is the effective area of the membrane, ∆*t* is the time interval, and ∆*P* is operating pressure. The total effective area of the membrane used in the dead-end permeation cell was 14.62 cm^2^.

The solute rejection, R (%), of the membrane against 1000 ppm of single solute was then calculated according to Equation (3) based on the conductivity measurement. The conductivity of the samples was first determined by a conductivity meter followed by concentration conversion based on the conductivity against the salt concentration calibration curve.

(3) $$R=\frac{c_{f}-c_{p}}{c_{f}}\times 100$$

where *c_f_* and *c_p_* are the concentrations of feed and permeate, respectively. The salt permeability, B, of a membrane was calculated according to the following equation:

(4) $$B=\left(\frac{1}{R}-1\right)\times A\left(\Delta P-\Delta \pi \right)$$

where *A*, Δ*P*, and Δ*π* in this equation refer to water permeability, operating pressure, and osmotic pressure of the feed solution, respectively.

For the FO/PRO experiment, a crossflow filtration setup equipped with two high-precision micro-gear pumps (WT3000-1JA, LongerPump, Hebei, China) was used. The total effective area of the membrane tested in the crossflow system was 42 cm^2^. The velocity of the feed and draw solution streams was circulated at 32.72 cm/s using two separate gear pumps. The draw solution tank with a 2 L maximum capacity was placed on a digital weight balance and the change in the draw solution was used to determine water flux.

The water flux of the same membrane under the same feed and draw solution is different, depending on the membrane orientation mode, i.e., FO mode (active-layer-facing-feed-solution, AL-FS) and PRO mode (active-layer-facing-draw-solution, AL-DS). Unless otherwise specified, the water flux of each experiment reported in this work was the average of three replications with a running time of 20 min each. In the case where DI water was used as the feed solution, only selected draw solutes were considered in this work. After that, the DI water feed solution was replaced with AT-POME and the performance of membranes was further characterized with respect to water flux, *J_v_* (kg/m^2^ h), and reverse draw solute flux, *J_s_* (g/m^2^ h), using the selected draw solute under FO/PRO mode. Both *J_v_* and *J_s_* can be determined using Equations (5) and (6), respectively.

(5) $$J_{v}=\frac{\Delta V}{A_{m}\cdot \Delta t}=\frac{\Delta m/\rho }{A_{m}\cdot \Delta t}$$

(6) $$J_{s}=\frac{C_{t}V_{t}-C_{0}V_{0}}{A_{m}\times \Delta t}$$

where ∆*V* is the volume difference of the draw solution, ∆*m* is the weight difference of the draw solution, *ρ* is the density of the feed solution, *C_t_* and *C*_0_ are the final and initial concentrations of the feed, respectively, and *V_t_* and *V*_0_ are the final and initial volumes of the feed solution, respectively.

Upon completion of the experiment, the performance of membranes was further analyzed by varying the crossflow velocity of the feed and draw solution streams. The performance of membranes was evaluated prior to *J_v_* and *J_s_* using the selected draw solute under FO/PRO mode.

A UV-vis spectrophotometer (DR5000, Hach) was used as an instrument to analyze the color intensity of AT-POME sample before and after filtration. The removal of color, R_c_ (%), was calculated using Equation (7):

(7) $$R_{c}=\left(1-\frac{{\mathrm{Abs}}_{\mathrm{permeate}}}{{\mathrm{Abs}}_{\mathrm{feed}}}\right)\times 100$$

where Abs_feed_ and Abs_permeate_ are the absorbances of the feed and permeate, respectively.

In order to measure the TOC value of the treated AT-POME sample, a TOC analyzer (TOC-LCPN, Shimadzu) was used. First, the sample liquid was injected into the 680 °C combustion tube to convert total carbon (TC) to CO_2_ for TC analysis. The sample that contained inorganic carbon (IC) was mixed and reacted with 1-N hydrochloric acid to produce carbon dioxide. The TOC was obtained by subtracting the TC value from the IC value. The TOC removal, R_TOC_ (%), was calculated using Equation (8):

(8) $$R_{c}=\left(1-\frac{{\mathrm{TOC}}_{\mathrm{permeate}}}{{\mathrm{TOC}}_{\mathrm{feed}}}\right)\times 100$$

where TOC_feed_ and TOC_permeate_ are the TOCs of the feed and permeate, respectively.

## 3. Results and Discussion

### 3.1. Performance Evaluation of Synthesized Thin-Film Composite Membranes

#### 3.1.1. Properties of PAN/PPSU Substrate and Its Impact on TFC Membrane

The effects of the PAN/PPSU substrate on the filtration performances of TFC membranes were evaluated with respect to water permeability and salt rejection using a dead-end filtration cell. The rejection experiment is important in order to confirm that the developed membranes are in the NF category, i.e., high rejection rates against divalent salts. A high rejection can ensure the membrane achieves minimum reverse solute flux during the FO or PRO process. In addition, it is also important to make sure the developed membranes are effective to remove color pigment (lignin and phenolics) from the AT-POME.

**Figure 2:**
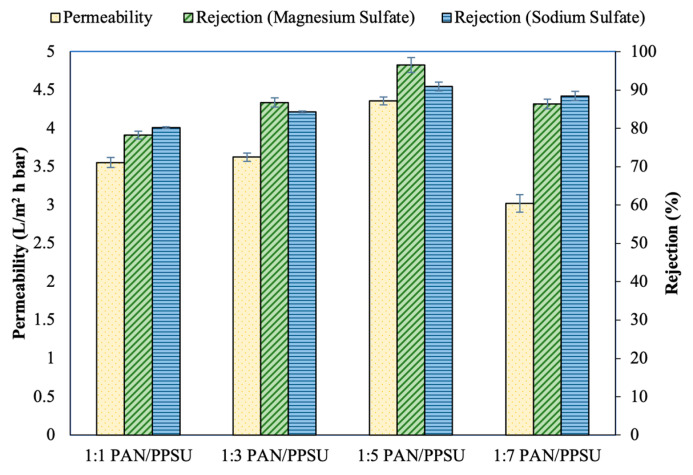
Permeability and salt rejection of TFC membranes made of different types of PAN/PPSU substrates without non-woven fabric (Operation mode: pressurized filtration; feed solution: 1000 ppm salt solution; operating pressure: 10 bar; temperature: 24 °C (+1)).

Figure 2 compares the pure water permeability and solute rejection of TFC NF membranes made of different substrates in filtering 1000 ppm of MgSO_4_ and Na_2_SO_4_ solution at 10 bar. As shown, the TFC membrane made of 1:5 PAN/PPSU substrate was the only membrane meeting the NF requirement, exhibiting >90% rejection against MgSO_4_ and Na_2_SO_4_. The rejection trends of the membrane against MgSO_4_ were in the order of 1:5 PAN/PPSU (96.48%) > 1:3 PAN/PPSU (86.73%) > 1:7 PAN/PPSU (86.32%) > 1:1 PAN/PPSU (78.22%), while the rejection trends against Na_2_SO_4_ were in the order of 1:5 PAN/PPSU (90.92%) > 1:7 PAN/PPSU (88.43%) > 1:3 PAN/PPSU (84.30%) > 1:1 PAN/PPSU (80.13%). With respect to water permeability, the TFC membrane made of 1:5 PAN/PPSU substrate was found to have a higher water flux (4.06 L/m^2^ h bar) compared to other synthesized membranes.

It must be noted that the pure PAN and PPSU polymer membranes were not fabricated and tested in this study. This is because PPSU is very soluble in NMP in which it tends to form gel except at very low concentration (<15 wt%) [26]. In addition, the active PA layer is also very easy to detach from the pure PPSU substrate, and this makes the resultant membrane not practical for use. According to a previous study [26], the PPSU membrane exhibits a large pore size and has a high fraction of macro-voids, which could decrease the effective porosity of the membrane. To cope with these problems, PPSU was blended with the PAN owing to the capability of PAN in forming covalent and ionic bonds with the amine compound in the PA layer upon surface modification using the NaOH solution [27]. The ionic bond between the two layers (PA and PAN) will improve the chemical stability of the skin layer with the blend substrate.

**Figure 3:**
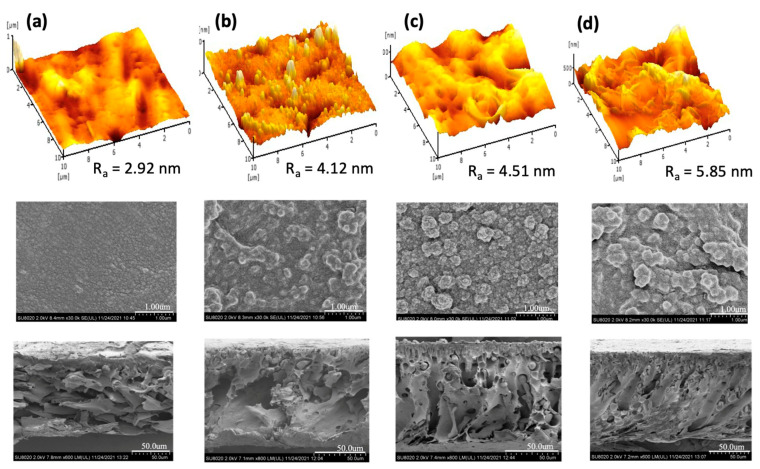
Three-dimensional AFM images (**top**), FESEM surface images (**middle**), and FESEM cross-sectional substrate images of TFC membrane made of different substrates: (**a**) 1:1 PAN/PPSU, (**b**) 1:3 PAN/PPSU, (**c**) 1:5 PAN/PPSU, and (**d**) 1:7 PAN/PPSU.

Figure 3 compares the surface morphology and cross-sectional structure of the TFC membrane made of different types of PAN/PPSU substrate. As can be seen, the PA and substrate properties of the membranes were altered by increasing the amount of PPSU in the PAN substrate. At a PAN/PPSU ratio of 1:1, the surface roughness (R_a_) of the resultant membrane was lowest among the four membranes fabricated. The result was in good agreement with the observation found on the FESEM surface image. Nevertheless, the cross-sectional structure of the substrate is not highly desired, as it exhibits high tortuosity, which is not favorable for water transport. This could be due to the unstable thermodynamic during the phase inversion process resulting from the equal ratio of highly hydrophilic PAN and semi-hydrophilic PPSU. A further increase in PPSU concentration from a PAN/PPSU ratio of 1:3 to 1:7 showed an increasing number of small nodules within the cross-sectional substrate and this directly increased the substrate roughness.

From the figure, the R_a_ value of the TFC membrane increased from 4.12 to 5.85 nm by varying the PAN/PPSU ratio from 1:3 to 1:7. It was found that at the highest PAN/PPSU ratio of 1:7, the membrane suffered from the lowest water permeability and salt rejection, and this could be due to the reduced compatibility of the two polymers when PPSU presented in large quantity. The reduced permeability could be caused by the pore blockage by the nodules, while the reduced salt rejection was due to the defect of the PA layer formed on the rough PAN/PPSU substrate. From the results, it can be seen that the surface morphology of the membrane made of 1:5 PAN/PPSU substrate was quite homogenous and its PA structure (PIP-TMC) was similar to that reported elsewhere [28,29]. Compared to the 1:3 PAN/PPSU substrate that exhibited large microvoids, the structure of the 1:5 PAN/PPSU substrate that was composed of short finger-like structures supported by a mixed porous morphology could offer better mechanical resistance.

**Figure 4:**
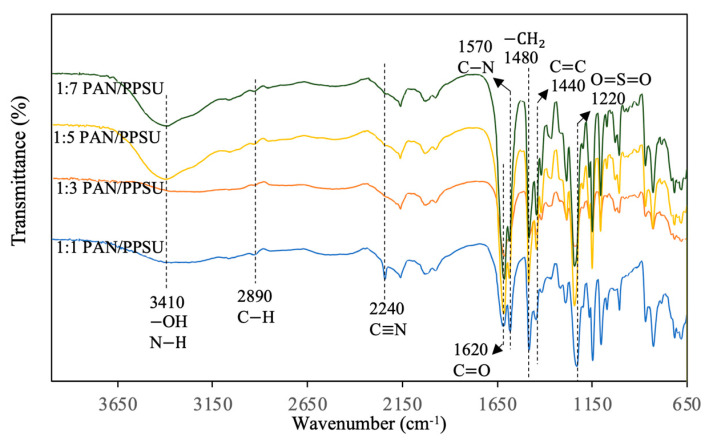
FTIR spectra of TFC NF membranes made of different substrates.

Figure 4 shows the FTIR spectra of the TFC membrane made of different PAN/PPSU substrates. The presence of PAN was confirmed by two peaks at 2890 and 2240 cm^−1^, which are attributed to C-H and C≡N bonds, respectively [30]. These two peaks were detected in the PAN/PPSU substrate that was composed of the highest amount of PAN, i.e., 1:1 PAN/PPSU. The peaks belonging to the PPSU were found at 1440 and 1220 cm^−1^ and they are assigned to C=C and O=S=O of the polymer, respectively [31]. It can be seen that the C≡N bond (2240 cm^−1^) disappeared when the amount of PPSU was increased from PAN/PSSU of 1:1 to 1:3, and this could be due to the insensitivity of FTIR in detecting small amounts of PAN presented in the PAN/PPSU substrates. The typical characteristics of PA that are the N-H streching band of PIP and the C=O streching band for the amide group can also be detected at ~3410 and 1620 cm^−1^, respectively [32]. As the PPSU polymer does not react chemically with PA layer, the use of modified PAN (containing −COOH groups after simple NaOH treatment) could improve the interation between the surface and PA formed via interfacial polymerization [27]. The −COOH groups of modified PAN could form covalent and ionic bonds with the amine compounds.

Although PAN is well-known for its highly hydrophilic and low fouling properties, the performance of the membrane made of pure PAN has been associated with brittleness and pore collapse during the drying process [33]. In contrast, PPSU is an amorphous thermoplastic polymer, which was reported to possess good mechanical strength and outstanding thermal and oxidative resistance [34]. Nevertheless, PPSU is more hydrophobic compared to the PAN. Therefore, with respect to surface hydrophilicity, it was found that the WCA of the membrane was increased by increasing the composition of PPSU in the substrate (see Table 3). The WCA trend of the substrate was gradually increased in the order of 1:1 PAN/PPSU (31.4°) < 1:3 PAN/PPSU (33.5°) < 1:5 PAN/PPSU (38.8°) < 1:7 PAN/PPSU (45.2°). The trend is similar to the WCA of the active PA layer formed atop the substrate, i.e., 1:1 PAN/PPSU (40.5°) < 1:3 PAN/PPSU (42.4°) < 1:5 PAN/PPSU (48.4°) < 1:7 PAN/PPSU (55.2°). It can be concluded that although the hydrophilicity of the substrate was adversely affected upon the incorporation of PPSU, the resultant PAN/PPSU substrate was better for the filtration process owing to the better mechanical and chemical properties of PPSU.

#### 3.1.2. Pressure-Driven Filtration of Wastewater Using Thin-Film Composite Membranes

**Figure 5:**
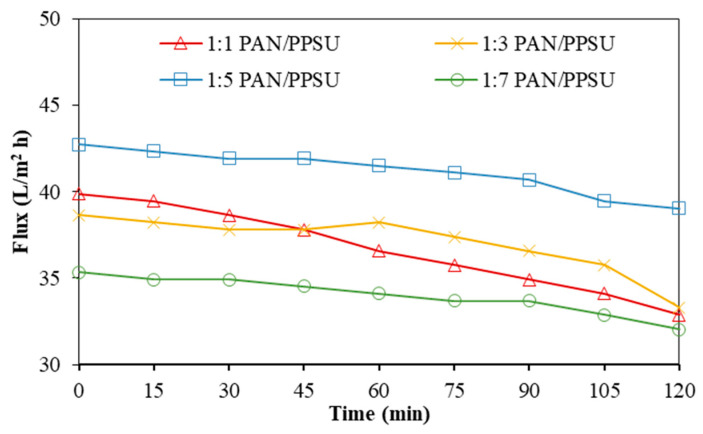
Water flux profile of TFC NF membranes made of different PAN/PPSU substrates as a function of filtration time. (Operation mode: pressurized filtration; feed solution: AT-POME; operating pressure: 10 bar; temperature: 24 °C (+1)).

The performances of the TFC NF membrane in treating AT-POME were evaluated and the results are presented in Figure 5. All of the self-fabricated TFC NF membranes suffered from flux deterioration as a function of filtration time. The decreased water flux can be explained as follows. First, the use of a dead-end filtration cell tended to increase the feed concentration when the permeate was produced. This, as a result, increased the osmotic pressure and reduced water flux. Secondly, the deposition of organic foulants during AT-POME filtration created additional resistance toward water transport, which led to lower flux. At the end of the 2 h operation, it was found that the membrane made of the 1:5 PAN/PPSU substrate attained the highest final water flux compared to the rest of the membranes. The membrane made of 1:7 PAN/PPSU meanwhile showed the lowest value. The water flux of AT-POME filtration was similar to the trend of the membrane’s pure water permeability, as shown in Figure 2.

The membrane separation performance during AT-POME treatment was further evaluated and the results are summarized in Table 4. All the TFC NF membranes showed good results in removing color components from AT-POME. The color removal rates were reported in the range of 90.3–97.8% in which the TFC membrane made of the 1:5 PAN/PPSU substrate exhibited slightly better removal rates. The membrane with an excellent color removal rate is highly recommended in order to ensure no color component could pass through the membrane to the draw solution during the FO/PRO process. If the color component does permeate from the feed solution to draw solution, it could defeat the purpose of using the FO/PRO process to treat AT-POME. The permeate sample was further analyzed with respect to TOC and conductivity. Compared to the TFC membrane made of the 1:5 PAN/PPSU substrate, the membrane made of the 1:7 PAN/PPSU substrate demonstrated even higher color and TOC removal rates even though its conductivity (corresponded to the dissolved ions) was lower. The only possible explanation for this result is mainly due to its roughest surface, which plays a more effective role in trapping organic foulants, preventing them to pass through the membrane (see Figure 3).

### 3.2. Evaluation of TFC Membrane Incorporating Coating Layer

#### 3.2.1. Effect of Coating on the Substrate Properties

**Figure 6:**
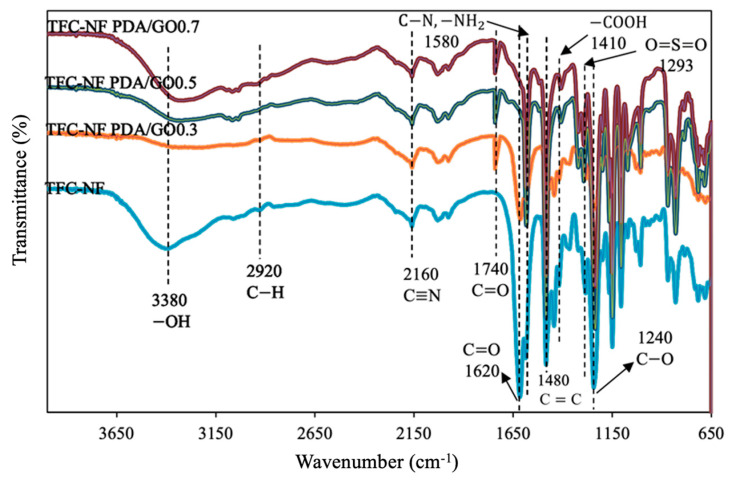
FTIR spectra of TFC NF membranes coated with different GO loading.

In this section, we studied the impact of the PDA/GO coating on the bottom substrate of the TFC membrane made of the best performing substrate, i.e., 1:5 PAN/PPSU. Figure 6 shows that the TFC NF membrane without the PDA/GO coating exhibited the key characteristics of PAN (i.e., 2890 cm^−1^ (C−H), 2240 cm^−1^ (C≡N), and 1480 cm^−1^ (−CH_2_)) and PPSU (i.e., 1440 cm^−1^ (C=C) and 1220 cm^−1^ (O=S=O)). The presence of PDA in the coating layer could be confirmed by the increased peak intensities of ketone (C=O, 1740 cm^−1^) and primary amine (−NH_2_, 1580 cm^−1^) [35,36,37]. Meanwhile, the peaks belonging to the GO could be categorized into the stretching band of hydroxyl (−OH, 3380 cm^−1^), ketone (C=O, 1740 cm^−1^), carbonyl (C=O, 1620 cm^−1^), carboxylic (−COOH, 1410 cm^−1^), and epoxy (C−O, 1240 cm^−1^) [38,39]. Some significant changes in the FTIR bands could be noticed when the membrane was coated with PDA/GO at varying GO loading. The peak at 3380 cm^−1^ (−OH functional group) in the coating layer of PDA/GO0.3 disappeared due to the reaction of carboxylic acid in GO with amine and the hydroxyl group in PDA. The result is in good agreement with other literature [40,41,42]. By further increasing the GO loading to PDA/GO0.5 and PDA/GO0.7, the intensity of the broad band between 3000 and 3600 cm^−1^ was increased owing to the presence of a significant amount of −OH groups from the use of high GO loading.

**Figure 7:**
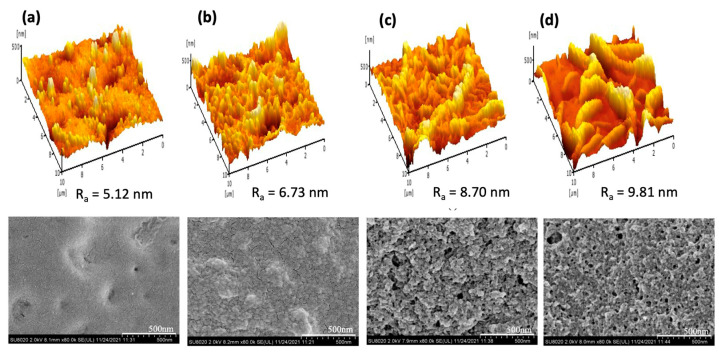
Three-dimensional AFM images (**top**) and FESEM images (**bottom**) of bottom surface of substrates with and without coating: (**a**) TFC NF (control), (**b**) PDA/GO0.3, (**c**) PDA/GO0.5, and (**d**) PDA/GO0.7.

Figure 7 compares the bottom surface of the TFC membrane with and without the coating. As shown, the bottom surface of the TFC membrane without the coating had a relatively small R_a_ value (4.12 nm) compared to other membranes with the substrate having an additional coating layer. The R_a_ value was gradually increased from 5.12 to 9.18 nm by increasing the amount of GO from zero to 0.7 g/L in the coating layer. The results revealed the impact of GO loading in increasing the roughness of the bottom surface. From the FESEM images, it was found that the coating was interconnected and dispersed homogeneously on the bottom surface when the membrane was coated with PDA/GO0.5. However, by increasing the GO loading to 0.7 g/L, severe nanoparticles aggregation was detected, which formed a rough layer during self-polymerization of PDA [43,44]. This self-polymerization made the polymer deposit unevenly on the surface of the substrate. Furthermore, aggregated GO is likely to block open pores on the bottom surface, which could negatively affect water flux. At the lowest GO loading (PDA/GO0.3), there was no obvious agglomeration on the resultant membrane surface, and this could be due to the formation of a relatively thin PDA/GO coating layer.

#### 3.2.2. Effect of Coating on the Thin-Film Composite Membrane Performance

##### Pressure-Driven Filtration of Wastewater

**Figure 8:**
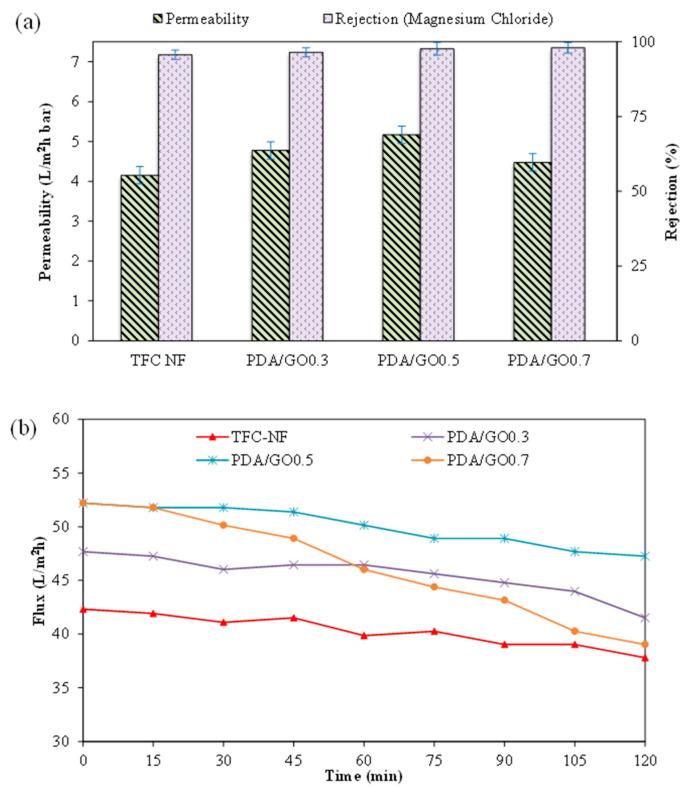
Performance of TFC membrane (made of 1:5 PAN/PPSU substrate) with and without PDA/GO coating layer in terms of (**a**) permeability and MgCl_2_ rejection (Operation mode: pressurized filtration; feed solution: 1000 ppm salt solution; operating pressure: 10 bar; temperature: 24 °C (+1)), and (**b**) water flux profile as a function of AT-POME filtration time (Operation mode: pressurized filtration; feed solution: AT-POME; operating pressure: 10 bar; temperature: 24 °C (+1)).

Figure 8a shows the pure water permeability and MgCl_2_ rejection of the TFC NF membrane with and without the PDA/GO coating layer at different GO loading. As the GO concentration in the PDA solution was increased from zero to 0.5 g/L, the membrane permeability was found to increase accordingly. The permeability, however, was reduced when the highest GO concentration (0.7 g/L) was used. In brief, the TFC NF membrane with the PDA/GO0.5 coating showed the highest permeability (5.16 L/m^2^ h bar) followed by PDA/GO0.3 (4.77 L/m^2^ h bar), PDA/GO0.7 (4.47 L/m^2^ h bar), and the control TFC membrane (4.15 L/m^2^ h bar). The results indicated that the hydrophilicity due to the PDA/GO coating was compromised when excessive GO was introduced into the coating solution, and this caused nanosheet agglomeration and reduced its effectiveness in promoting water transport. With respect to salt rejection, it was found that all the membranes demonstrated very similar separation efficiency. This is because the coating layer was only performed on the bottom surface of the substrate, and the PA layer that acted as a selective layer remained intact.

Figure 8b shows that the membrane with the PDA/GO0.7 coating layer suffered the most severe flux decline (~25%) during 2 h AT-POME filtration. As a comparison, the TFC membrane without the coating layer recorded a <10% flux decline. It is quite clear that the presence of a large amount of GO in the coating layer could lead to increased water transport resistance, which reduced water flux. For the membrane coated with 0.5 g/L of GO (i.e., PDA/GO0.5), the flux decline was quite minimal (<10%). In addition, the presence of the PDA/GO layer offered a great improvement for the membrane water flux during AT-POME treatment. At the end of the experiment, all the coated membranes, in particular, the membrane coated with 0.5 g/L of GO, showed higher water flux compared to the control TFC membrane.

Table 5 presents the water contact angle of the TFC membrane with and without the PDA/GO coating layer. It was proved that the PDA/GO coating layer could improve the hydrophilicity of the substrate’s bottom due to the hydrophilic characteristics of GO. The bottom surface of the pristine TFC NF membrane was 35.2°. When the PDA/GO was coated on the substrate, the contact angle of the substrate was decreased. By increasing the GO concentration from 0.3 to 0.5 g/L in the coating solution, the contact angle of the membrane was decreased from 28.8° to 21°. The contact angle, however, was not further reduced by increasing the GO concentration to 0.7 g/L, owing to the agglomeration of nanosheets, which compromised the good features of GO.

The membrane separation performance during AT-POME treatment is summarized in Table 6. As can be seen, promising results were obtained for the color removal rates regardless of the types of membranes. The color removal rates were reported in the range of 95.32–97.93%. The results are reasonable as the filtration process was carried out in the dead-end mode and the modification of the substrate’s bottom properties would not affect greatly the membrane rejection rate.

##### Engineered Osmosis Filtration of Wastewater

**Figure 9:**
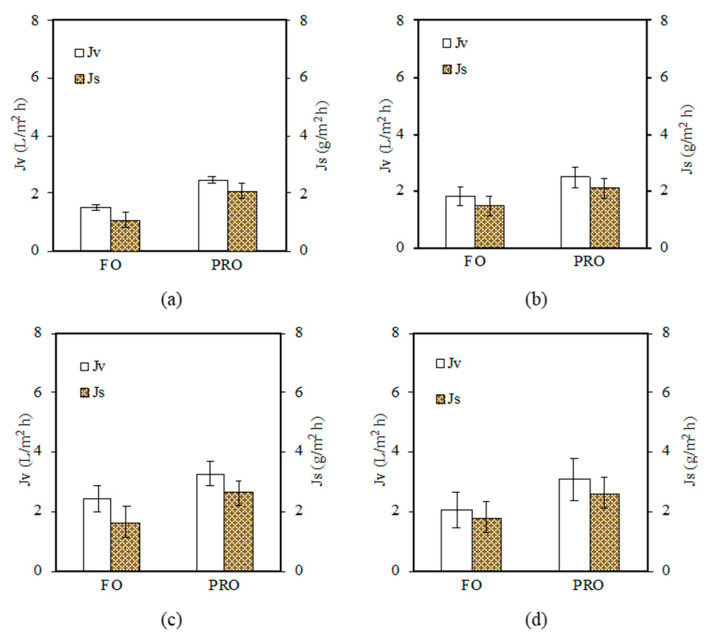
Water flux (*J_v_*) and reverse solute flux (*J_s_*) of TFC NF membranes under FO and PRO mode: (**a**) TFC NF (control), (**b**) PDA/GO0.3, (**c**) PDA/GO0.5, and (**d**) PDA/GO0.7 (Operation mode: engineered osmosis; feed solution: pure water; draw solution: 4 M MgCl_2_; cross flow velocity: 0.3272 m/s for both feed and draw solution; operation period: 2 h; temperature: 24 °C (+1)).

The performances of the TFC membrane with different PDA/GO coating layers were further tested in the FO/PRO mode using pure water as the feed solution, and the results are presented in Figure 9. The significant water flux increase was observed when the GO concentration of 0.5 g/L was introduced. This membrane (PDA/GO0.5) achieved 2.41 L/m^2^ h in FO mode and 3.26 L/m^2^ h in PRO mode. Meanwhile, the lowest water flux was recorded by the TFC membrane with the PDA/GO0.7 coating layer (FO mode: 2.03 L/m^2^ h; PRO mode: 3.08 L/m^2^ h). By introducing the PDA/GO coating layer onto the bottom surface of the substrate, the water molecule could transport across the membrane faster and reduce reverse solute flux. The PDA/GO layer was interconnected and homogeneously formed on the substrate; therefore, it could promote the efficient exchange of water and salt across the membrane. This could lead to a milder internal concentration polarization (ICP) effect and improved water permeation [30].

The presence of a hydrophilic layer on the bottom surface of the substrate is effective against ICP as it allows a complete wetting of the substrate, which improves its wettability and decreases effective tortuosity [18,45]. Clearly, the use of an appropriate amount of GO in the coating layer could further improve the performances of the TFC NF membrane. For all the TFC membranes, similar reverse solute fluxes were obtained, implying that the presence of the PDA/GO layer on the bottom surface of the membrane did not significantly affect the selectivity of the PA layer.

**Figure 10:**
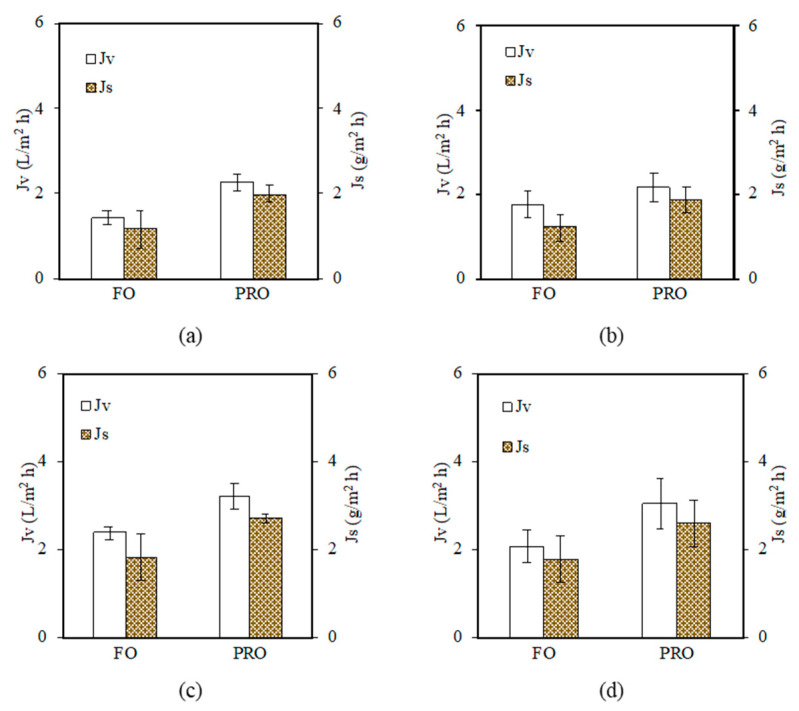
Water flux (*J_v_*) and reverse solute flux (*J_s_*) of TFC NF membrane under FO and PRO mode: (**a**) TFC NF (control), (**b**) PDA/GO0.3, (**c**) PDA/GO0.5, and (**d**) PDA/GO0.7 (Operation mode: engineered osmosis; feed solution: AT-POME; draw solution: 4 M MgCl_2_; cross flow velocity: 0.3272 m/s for both feed and draw solution; operation period: 2 h; temperature: 24 °C (+1)).

Figure 10 compares the performance of different TFC membranes in treating AT-POME under two different osmotic processes. It was found that the TFC membrane with the PDA/GO0.5 coating layer exhibited the most promising results in both FO and PRO processes. Its water flux and reverse solute flux in the FO process were 2.37 L/m^2^ h and 1.80 g/m^2^ h, respectively. Meanwhile, its PRO performance showed a water flux of 3.20 L/m^2^ h and reverse solute flux of 2.70 g L/m^2^ h. The water flux of the PRO process was 35% higher compared to the FO process. Compared to the pristine TFC membrane (without coating), the TFC membrane with the PDA/GO0.5 coating layer recorded 67% and 41% higher FO and PRO water flux, respectively, during AT-POME treatment. The results clearly indicated the positive impacts of the hydrophilic surface coating on the FO/PRO performance of the TFC NF membrane in treating AT-POME.

As summarized in Table 7, it can be seen that the self-fabricated TFC NF membrane coated with the PDA/GO layer could outperform the commercial NF90 and NF270 membranes in both FO mode and PRO mode with respect to water flux. The water flux of the self-fabricated TFC NF membrane was observed to have quadrupled compared to the commercial membranes. It could be concluded that the membrane coated with the PDA/GO membrane had good stability and performances in either the pressure-driven filtration process or engineered osmosis process due to the excellent properties of the PAN/PPSU substrate coupled with the appropriate hydrophilic PDA/GO coating. Furthermore, the elimination of thick non-woven fibers during TFC membrane fabrication was effective in reducing the ICP effect during the FO/PRO process, offering minimal water transport resistance for the membrane.

## 4. Conclusions

In this work, we have successfully demonstrated that modifying the substrate properties of the TFC membrane could lead to improved membrane performance for AT-POME treatment via the engineered osmosis process. Our findings showed that using the blend substrate at a PAN/PPSU weight ratio of 1:5 (without having thick non-woven support) could produce the TFC membrane with the highest water flux and divalent salt rejection compared to the membranes made of different substrates at varying PAN/PPSU ratios. The improved properties could be due to the relatively good compatibility between PAN and PPSU at this ratio, which formed a substrate with short finger-like structures supported by a mixed porous morphology. Compared to the membrane made of the highest PAN/PPSU ratio (1:7), the best performing TFC membrane made of a PAN/PPSU ratio of 1:5 exhibited a smoother surface and had homogenous PA morphology. Furthermore, its cross-sectional structure was not blocked by the nodules as found in the membrane made of a PAN/PPSU ratio of 1:7. By forming an additional hydrophilic layer (PDA/GO0.5) on the bottom surface of the best PAN/PPSU substrate, the resultant membrane demonstrated improved water flux while maintaining similar divalent salt rejection. The improved water flux is due to the improved membrane hydrophilicity resulting from the hydrophilic coating layer. It must be pointed out that the presence of a hydrophilic layer on the bottom surface of the substrate is effective against ICP during the engineered osmosis process as it allows a complete wetting of the substrate. When tested using AT-POME as a feed solution and 4 M MgCl_2_ as a draw solution, the best performing TFC membrane with the hydrophilic coating layer achieved 67% and 41% higher FO and PRO water flux, respectively, compared to the TFC membrane without having a coating layer. More importantly, the TFC membrane with the hydrophilic coating layer could attain very high color rejection (>97%) during AT-POME treatment, and its performance (water flux and reverse solute flux) during the engineered osmosis process was even better compared to the commercial NF90 and NF270 membranes. The promising outcomes were attributed to the excellent properties of the PAN/PPSU substrate that was coated with a hydrophilic PDA/GO coating and the elimination of thick non-woven fiber during TFC membrane fabrication to reduce the ICP effect during the FO/PRO process.

## Figures and Tables

**Table 1:** Characteristics of the AT-POME.

Parameter	Conductivity (μS)	Color (ADMI)	Color (Abs)	TOC (ppm)
Value	7855 (±37.50)	1635 (±0.06)	2.64 (±0.20)	162.33 (±0.47)

**Table 2:** Dope formulation for PAN/PPSU substrates fabrication.

PAN/PPSU Ratio	PAN (wt%)	PPSU (wt%)	NMP (wt%)
1:1	8.0	8.0	84.0
1:3	4.0	12.0	84.0
1:5	2.7	13.3	84.0
1:7	2.0	14.0	84.0

**Table 3:** Surfaces contact angle of TFC membranes.

Types of TFC Membrane	Contact Angle (°)	
	Top Active Layer	Bottom Layer
1:1 PAN/PPSU	40.54 (±2.26)	31.43 (±1.56)
1:3 PAN/PPSU	42.45 (±2.16)	33.54 (±1.86)
1:5 PAN/PPSU	48.44 (±2.19)	38.83 (±2.26)
1:7 PAN/PPSU	55.24 (±2.36)	45.23 (±2.66)

**Table 4:** Comparison between the separation performances of TFC NF membranes in the AT-POME treatment under pressure-driven mode.

Parameter	Removal (%)			
	1:1 PAN/PPSU	1:3 PAN/PPSU	1:5 PAN/PPSU	1:7 PAN/PPSU
Conductivity	23.51 (±1.26)	31.36 (±1.26)	42.71 (±1.26)	40.78 (±1.26)
Color (ADMI)	90.26 (±1.26)	94.09 (±1.26)	96.55 (±1.26)	97.79 (±1.26)
Color (Abs)	93.30 (±1.26)	94.58 (±1.26)	96.73 (±1.26)	97.73 (±1.26)
TOC	63.55 (±1.26)	65.09 (±1.26)	73.53 (±1.26)	73.63 (±1.26)

**Table 5:** Surface contact angle of bottom layer of TFC membrane with and without PDA/GO coating layer.

Types of TFC Membrane	Contact Angle (°)
TFC NF	35.2 (±1.56)
PDA/GO0.3	28.8 (±1.86)
PDA/GO0.5	21.4 (±2.26)
PDA/GO0.7	23.5 (±2.66)

**Table 6:** Comparison between the separation performances of TFC NF membranes with and without PDA/GO coating layer for AT-POME treatment.

Parameter	Removal (%)			
	TFC NF	PDA/GO0.3	PDA/GO0.5	PDA/GO0.7
Color (ADMI)	95.95 (±1.72)	96.75 (±1.76)	97.37 (±1.79)	97.74 (±1.74)
Color (Abs)	95.32 (±1.38)	96.40 (±1.36)	97.19 (±1.59)	97.93 (±1.38)

**Table 7:** Performance comparison between commercial membranes and best performing TFC membrane developed in this work for AT-POME treatment in FO and PRO mode.

Membrane	Water Flux, *J_v_* (L/m^2^ h)		Reverse Solute flux, *J_s_* (g/m^2^ h)	
	FO Mode	PRO Mode	FO Mode	PRO Mode
Commercial NF90	0.47 (±0.31)	1.12 (±0.38)	0.48 (±0.16)	0.17 (±0.04)
Commercial NF270	0.23 (±0.17)	0.55 (±0.26)	0.37 (±0.24)	0.15 (±0.03)
TFC coated with PDA/GO0.5	2.41 (±0.31)	3.26 (±0.34)	1.80 (±0.14)	2.70 (±0.29)

## Data Availability

Data will be made available on request.

## Abbreviations

AT-POME	Aerobically treated palm oil mill effluent
BOD	Biological oxygen demand
COD	Chemical oxygen demand
CPO	Crude palm oil
FO	Forward osmosis
GO	Graphene oxide
ICP	Internal concentration polarization
NF	Nanofiltration
PA	Polyamide
PAN	Polyacrylonitrile/polyphenylsulfone
PAN/PPSU	Polyacrylonitrile/polyphenylsulfone
PDA	Polydopamine
PDA/GO	Polydopamine/graphene oxide
PIP	Piperazine
POME	Palm oil mill effluent
PPSU	Polyphenylsulfone
PRO	Pressure retarded osmosis
RO	Reverse osmosis
TFC	Thin-film composite
TMC	Trimesoyl chloride
TOC	Total organic carbon
TSS	Total suspended solid
UF	Ultrafiltration
